# Cardiovascular magnetic resonance imaging produces highly reproducible rodent cardiac volumetric and functional data using a 1.5 Tesla scanner

**DOI:** 10.1186/1532-429X-18-S1-P191

**Published:** 2016-01-27

**Authors:** Gayathri Kumarasinghe, Chung Yao Yu, Michael Chang, Kirsten Moffat, Rene M Botnar, James Otton, Cameron Holloway, Jane McCrohon, Peter Macdonald, Andrew Jabbour

**Affiliations:** 1 grid.1057.30000000094723971Victor Chang Cardiac Research Institute, Darlinghurst, NSW Australia; 2 grid.437825.f0000000091192677Dept of Radiology, St. Vincent's Hospital, Darlinghurst, NSW Australia; 3 grid.13097.3c0000000123226764Dept of Cardiovascular Imaging, The Rayne Institute, Kings College London, London, United Kingdom; 4 Dept of Cardiology, St. Vincent's Hospital, Darlinghrust, NSW Australia

## Background

Rodent models of cardiovascular disease provide a means of rapid assessment of disease processes and therapeutic interventions. Cardiac magnetic resonance (CMR) provides superior functional imaging over other modalities, however has technical challenges in rodents due to small hearts sizes and rapid heart rates. High field strength scanners are often required, however access is limited. We therefore aimed to determine the reproducibility of functional CMR imaging of rodents using a clinical scanner.

## Methods

Eight healthy Lewis rats underwent CMR imaging using a 1.5T scanner and a clinical wrist coil. Electrocardiograph-gated bSSFP cine imaging was used to acquire ventricular long axis, four chamber and short-axis views. End-diastolic volume (EDV), ejection fraction (EF) and myocardial mass (MM) were measured using short-axis stack images. After initial scanning, the rodents were taken off the scanner table, then immediately returned for repeat imaging. Intra- and inter-observer variability and inter-study reproducibility were assessed. Sample size calculations were performed to determine animal numbers needed to detect a 10% difference in cardiac volumes, EF or MM. Comparison was made with two-dimensional echocardiography using published data.

## Results

Image quality was invariably good (Figure). Excellent intra-observer reproducibility was observed: coefficients of variability (COV) 3.17%, 1.61% and 5.39% for EDV, EF and MM. Inter-observer variability was likewise good: COV were 7.88%, 5.88% and 10.91% respectively. Inter-study reproducibility was likewise high: COV 6.93%, 4.56% and 3.66% respectively. Measurement agreements were favourable compared to rodent CMR studies using high field-strength scanners and human CMR reproducibility studies. Measurement agreements were superior to rodent and human echocardiography. A calculated sample size reduction of 96% can be achieved to detect a 10% change in MM using 1.5T CMR compared with two-dimensional echocardiography, with reductions in sample sizes by 65% and 78% to detect a 10% change in EDV and EF respectively.

## Conclusions

Accurate volumetric and functional data from healthy rodent hearts are obtainable using a clinical 1.5T scanner. The application of this protocol will allow wider access to CMR imaging in small rodent cardiovascular disease and therapeutic models with significant reduction in sample size to allow more accurate volumetric and functional measurements compared to echocardiography.

**Figure 1:**
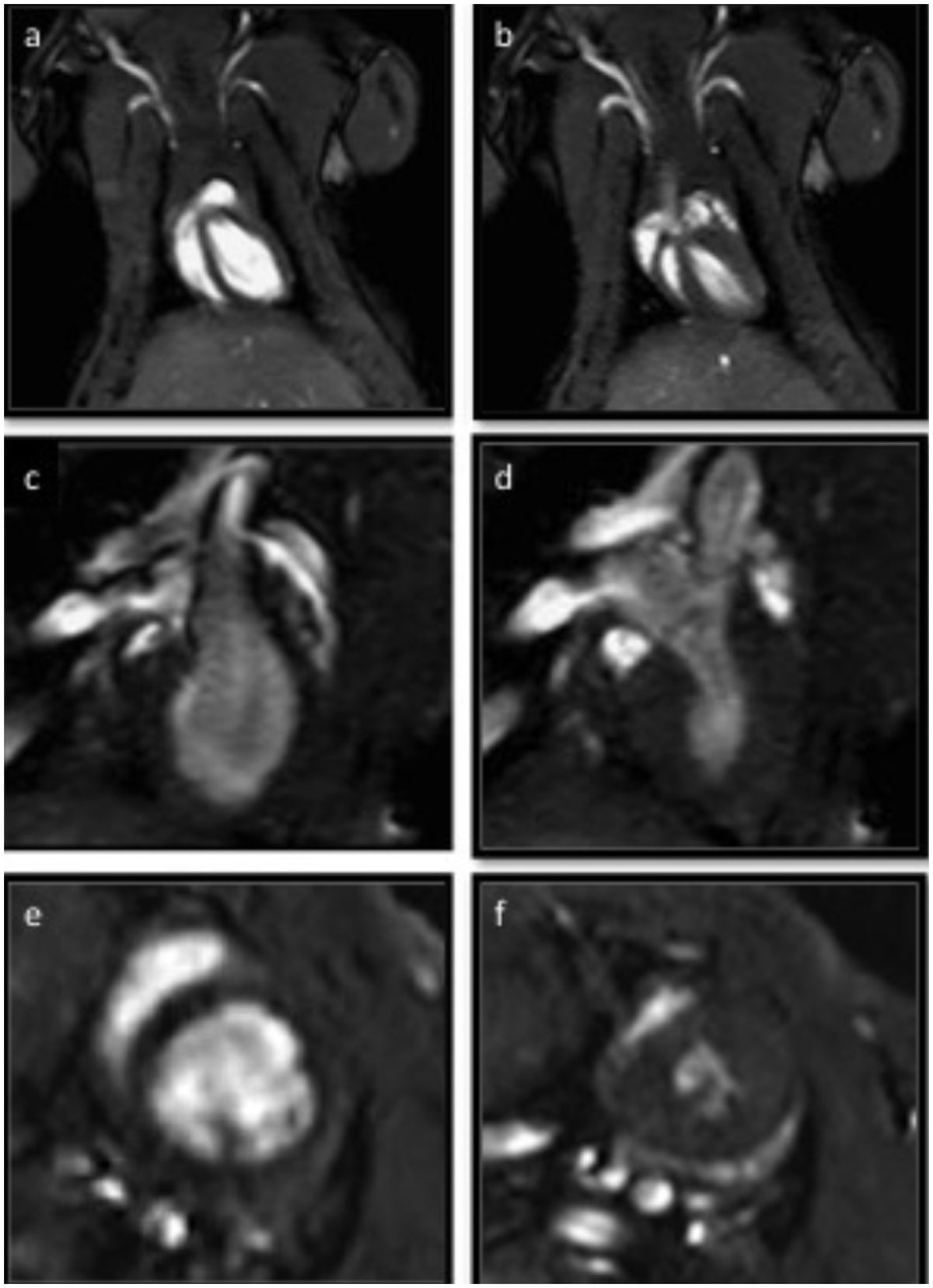
**Images of rat hearts in end-diastole and end-systole in ventricular four chamber (a and b), two chamber (c and d) and mid-ventricular short axis (e and f) views**.

